# Remote monitoring and teleconsultations can reduce greenhouse gas emissions while maintaining quality of care in cystic fibrosis

**DOI:** 10.3389/fdgth.2024.1469860

**Published:** 2024-10-24

**Authors:** Martinus C. Oppelaar, Michiel A. G. E. Bannier, Monique H. E. Reijers, Hester van der Vaart, Renske van der Meer, Josje Altenburg, Lennart Conemans, Bart L. Rottier, Marianne Nuijsink, Lara S. van den Wijngaart, Peter J. F. M. Merkus, Jolt Roukema

**Affiliations:** ^1^ Department of Pediatric Pulmonology, Radboud University Medical Center, Amalia Children’s Hospital, Nijmegen, Netherlands; ^2^ Department of Paediatric Pulmonology, MosaKids Children’s Hospital, Maastricht University Medical Centre+, Maastricht, Netherlands; ^3^ Department of Pulmonology, Radboud University Medical Center, Nijmegen, Netherlands; ^4^ Department of Pulmonary Diseases, University of Groningen, University Medical Center Groningen, Groningen, Netherlands; ^5^ Department of Pulmonology, Haga Teaching Hospital, The Hague, Netherlands; ^6^ Department of Respiratory Medicine, Amsterdam University Medical Centers, University of Amsterdam, Amsterdam, Netherlands; ^7^ Department of Respiratory Medicine, Maastricht University Medical Centre, Maastricht, Netherlands; ^8^ Division of Respiratory & Age-Related Health, Department of Respiratory Medicine, NUTRIM Institute of Nutrition and Translational Research in Metabolism, Maastricht, Netherlands; ^9^ Department of Pediatric Pulmonology and Pediatric Allergology, Beatrix Children’s Hospital, University of Groningen, University Medical Center Groningen, Groningen, Netherlands; ^10^ Groningen Research Institute for Asthma and COPD (GRIAC), University of Groningen, University Medical Center Groningen, Groningen, Netherlands; ^11^ Haga Teaching Hospital, Juliana Children’s Hospital, The Hague, Netherlands

**Keywords:** telemonitoring, cystic fibrosis, pediatrics, spirometry, telehealth, climate co-benefits, respiratory medicine

## Abstract

**Background:**

Remote care usefulness and climate change co-benefits should be addressed simultaneously to incentivize political action.

**Objectives:**

To assess the changes in healthcare consumption, lung function and greenhouse gas (GHG) emissions during the COVID-19 pandemic in Dutch cystic fibrosis (CF) care.

**Design:**

Retrospective multicentre observational study in five Dutch CF centres.

**Methods:**

Eighty-one participants were included. Healthcare consumption was described alongside the COVID-19 Stringency Index (2019–2022). Travel related GHG emissions were calculated for every clinic visit. Changes in percentage predicted Forced Expiratory Volume in one second (ppFEV1) were assessed using a paired-samples *T*-test.

**Results:**

Healthcare consumption patterns followed COVID-19 public health measure stringency but returned back to the “old normal”. Emission of 5.450, 3 kg of carbon dioxide equivalents were avoided while quality of care was relatively preserved. ppFEV1 declined as expected (*Δ*Means 3.69%, 95%CI 2.11–5.28).

**Conclusion:**

Remote monitoring of lung function and symptoms and teleconsultations in CF can reduce GHG emissions while maintaining quality of care. As health sectors constitute a large share of national climate change footprints, digital health can partly alleviate this burden by reducing private travel.

## Introduction

The COVID-19 pandemic dramatically changed the delivery of care to people with cystic fibrosis (pwCF) and people with chronic respiratory diseases in general. Whereas frequent physical outpatients visits were the standard for follow-up of pwCF, remote monitoring and teleconsultations became more prominent and necessary during the COVID-19 pandemic (1, 2). A recent multicenter study from the Netherlands showed that also after the COVID-19 pandemic over 75% of pwCF and healthcare professionals want more remote monitoring and teleconsultations and less in-person care in future, especially in adult CF care (3). The main reported reasons were improved physical conditions thanks to new modulator therapies, but also the possibility for pwCF to reclaim agency over their lives and to reduce the burden of hospital visits (3). It is argued that remote care could also reduce health system costs and improve healthcare professional labour efficiency, but undisputed evidence to support these claims are still scarce. However, similar findings from the United Kingdom and Sweden further illustrate how the perceptions of the CF care model are changing from a traditionally hospital-based model towards a more hybrid-care model (4, 5).

The question remains whether the CF care model itself is ready for these changes and what the effects of this would be on both CF care and the health of pwCF (1–3). In theory, there is also the potential for co-benefits of remote monitoring for climate change mitigation as hybrid care implies less private travel and hence lower greenhouse gas (GHG) emissions (6). By addressing these questions in unison we can create political will to accelerate the digital transition of healthcare. This brief report aimed to evaluate the changes in physical and remote healthcare consumption and the accompanying trends of GHG emissions due to private travel and lung function for pwCF before and during the COVID-19 pandemic.

## Methods

This was a retrospective observational multicenter study in five Dutch CF centres (Radboud university medical center, Maastricht university medical centre +, University medical centre Groningen, Haga Hospital, and Amsterdam university medical centres) and was part of a larger evaluation of remote monitoring in CF care (3). Eighty-one pwCF who used a remote monitoring programme (RMP) for their regular care during the COVID-19 period participated. The RMP consisted of home monitoring of lung function with portable spirometers and symptoms with questionnaires. It also facilitated safe and accessible patient-clinician contact. More details on the RMP including screenshots of the website and smartphone application can be found elsewhere (3). Study data were collected as part of regular care. Details on the informed consent and selection procedure, and approval from the ethical committee can be found in a previous publication (3).

The study period of interest was March 2019 until March 2022 and therefore divided in one pre-COVID-19 year (March 2019–February 2020) and two COVID-19 years (March 2020–February 2021; March 2021–February 2022). First, the total number of physical and teleconsultations in every month, as well as the total number of home and clinic spirometry tests during every month, were plotted against the COVID-19 stringency index of the Netherlands (7, 8). The COVID-19 Stringency Index is a composite score of nine metrics (school closures; workplace closures; cancellation of public events; restrictions on public gatherings; closures of public transport; stay-at-home requirements; public information campaigns; restrictions on internal movements; and international travel controls) that reflect overall strictness of public health COVID-19 measures with zero being least strict and hundred the most strict (7, 8). Teleconsultations were defined as any consultation using any communication medium (i.e., telephone or video) with the specific intent to replace a physical outpatient visit. Second, we assessed how many pwCF were able to meet quality criteria of four or more outpatient visits and clinic spirometry tests without and with remote monitoring for every year.

Third, we estimated the amount of GHG emissions expressed as kilogrammes of carbon dioxide equivalents (kgCO_2_eq) for every year based on private travel. The shortest home-hospital personal travel distance was calculated using the participants’ place of residence and treating CF centre for every outpatient clinic visit or clinic spirometry test using Google Maps. The total distance travelled per year was converted to kgCO_2_e by using the United Kingdom conversion factor of an average petrol care (0.17 kgCO_2_e/km) (9). Finally, safety of remote care was evaluated by examining the number of emergency department visits and inpatient stays over time, as well as the change in lung function. We performed a paired samples *t*-test with individuals’ highest percentage predicted forced expiratory flow in one second (ppFEV1) measured by clinic spirometry in the pre-COVID-19 and in the second COVID-19 year to evaluate changes in lung function during this period. Lung function tests conducted after individual's start date of the new modulator therapy Elexacaftor/Tezacaftor/Ivacaftor (ETI) were excluded.

## Results

Table 1 shows demographics of pwCF included in this study.

**Table 1:** Demographics of participants.

People with CF (*n* = 81)	
Male sex, *n* (%)	40 (49.4)
Age in 2020, median (IQR)	26 (12–40)
Age range in 2020	5–59
Number of pwCF per center, median (IQR)	14 (13–16)
*CFTR* genotype, *n* (%)	
F508del homozygous	49 (61)
F508del heterozygous	27 (33)
Other	5 (6)
Highest FEV1 in 2020, (*n* = 77)	
z-score FEV1, median (IQR)	−2.0 (−4.4 to −0.9)
FEV1 percentage predicted, median (IQR)	75.1 (58.2–89.6)

CFTR, cystic fibrosis transmembrane conductance regulator protein; FEV1, forced expiratory volume in one second.

**Figure 1:**
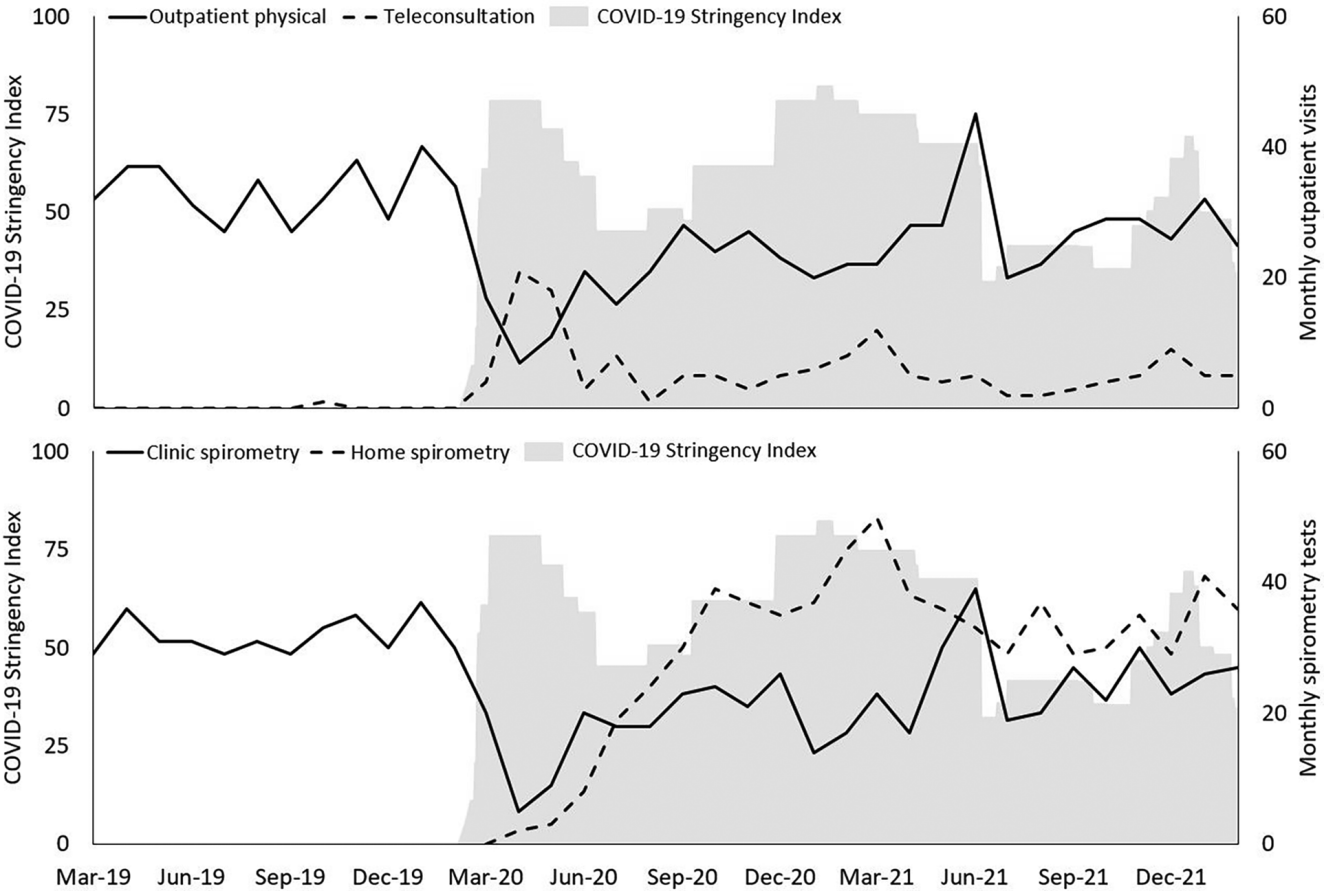
Patterns of healthcare consumption during every month plotted from march 2019 until march 2022 against the Dutch COVID-19 stringency Index (grey shaded area). Upper panel: Physical outpatient visits vs. teleconsultations. Lower panel: Clinic spirometry vs. home spirometry. Primary *Y*-axis represents COVID-19 Stringency Index value; secondary *Y*-axis represents healthcare consumption data.

Figure 1 shows the total number of monthly outpatients visits (physical vs. teleconsultations) and the total number of spirometry tests (clinic vs. home) over time. In the pre-covid year, only one teleconsultation had been performed across the 81 participants. During the first lockdown period the frequency of physical outpatient visits and clinic spirometry tests declined drastically whereas the frequency of teleconsultations suddenly reached an all-time high. After the first COVID-19 lockdown, the number of physical outpatients visits steadily increased back to normal with a peak when COVID-19 stringency measures were scaled down in summer 2021, potentially to catch-up lost visits. The number of teleconsultations declined steadily over time, with incidental peaks corresponding to peaks in COVID-19 stringency measures (i.e., lockdown periods). This emphasises that a paradigm shift regarding remote technologies in regular CF care had not yet occurred and that remote technologies were mostly used as a temporary replacement while regular physical care remained the norm.

In the pre-COVID-19 year, 77.8% and 75.3% of pwCF had four or more outpatient visits and four or more clinic spirometry tests respectively. In the two COVID-19 years, this number decreased to 30.9% in the first COVID-19 year and 51.9% in the second COVID-19 year for outpatient visits, and to 30.9% and 55.6% for clinic spirometry. When we include teleconsultations and home spirometry tests, 59.3% and 79.0% of pwCF had four or more outpatient visits and spirometry tests in the first COVID-19 year respectively, and 70.4% and 92.6% in the second year. Although remote technologies were mostly used as a temporary replacement, they effectively helped to preserve the follow-up of pwCF whereas this would not have been possible without them.

Travel distance was available for 80 of the 81 participants. Cumulative distance travelled and GHG emissions are presented in Table 2. Cumulative amount of GHG emissions was 41.7% lower in the first COVID-19 year and 22.7% lower in the second COVID-19 year compared to the pre-COVID-19 year. The total reduction in the amount GHG emissions over the two COVID-19 years was 5.450,3 kgCO_2_e which loosely amounts to 248 adult trees for reference (10). When extrapolating this number to the total number of pwCF in the Netherlands in 2022 (1.635), this would be 111.390,5 kgCO_2_e (or 5.068 trees) in just two years (10, 11). Although this calculation is incomplete and does not include all CO_2_ emissions (e.g., production of home spirometers; operation of the RMP; unused hospital capacity), it strongly suggests that remote technologies can both reduce travel distance, save time and costs for patients, but can also significantly reduce healthcare related GHG emissions.

**Table 2:** Distance travelled and greenhouse gas emissions (GHG) during the three study periods.

Period	Cumulative distance travelled	Median (IQR) distance travelled	Cumulative GHG emissions	Relative reduction from baseline GHG emissions
March 2019–February 2020	49,721.2 km	422.7 km (789.9)	8,476.5 kgCO_2_e	N/A
March 2020–February 2021	28,993.6 km	231.6 km (458.9)	4,942.8 kgCO_2_e	−41.7%
March 2021–February 2022	38,478.8 km	360.3 km (596.2)	6,559.9 kgCO_2_e	−22.7%

Pre-COVID-19 year: March 2019–February 2020; first COVID-19 year: March 2020–February 2021; second COVID-19 year: March 2021–February 2022. The pre-COVID-19 year is taken as the baseline measurement.

The total number of emergency department visits decreased from 24 in the pre-COVID-19 year to 11 and 16 in the first and second COVID-19 year respectively. Median (IQR) inpatient stays in the pre-COVID-19 year [21 (7–41), *n* = 22] also decreased in the first [9 (3–14), *n* = 17] and second post-COVID-19 year [11 (4–28), *n* = 19]. These findings suggest that there was no increase in pulmonary exacerbations requiring in-hospital care or that more problems could be treated remotely. These findings should be interpreted with caution as no information on antibiotic therapies nor microbiological assessments were available and because social distancing measures also reduced exposure to other pathogens than COVID-19. However, these findings do suggest that the increased remoteness of care did not have strongly adverse effects during this period and within this population.

Mean highest individual ppFEV1 was 76.3% (95%CI 70.9–81.8; *n* = 75) in the pre-COVID-19 year, 77.0% (95%CI 72.0–82.1; *n* = 68) in the first COVID-19 year, and 75.4% (95%CI 70.2–80.6; *n* = 70) in the second COVID-19 year. Paired samples *t*-test could be performed for 70 pwCF. On average, highest ppFEV1 was significantly lower in the second COVID-19 year compared to the pre-COVID-19 year [*Δ*Means 3.69%, 95%CI 2.11–5.28, t(69) = 4.66, *p* = <0.001, *r* = 0.49]. This decline is line with the natural reported progression of CF pulmonary disease which averages 1–3 percentage points ppFEV1 annually (12, 13). In contrast, some studies have found increasing or stable ppFEV1 values in pwCF during the COVID-19 pandemic (14, 15). These studies included general CF cohorts and our relatively small sample might have been subject to selection bias. Moreover, differences between national public health measures for COVID-19 could have impacted underlying determinants of pwCF's health across countries differently (e.g., possibility for physical activity). This should be studied in more detail and in more generalized cohorts.

## Discussion

Overall, the way in which healthcare for pwCF in the Netherlands was delivered changed drastically during the COVID-19 years. This resulted in reductions in travel time and GHG emission which was beneficial for empowering pwCF in their daily lives and had co-benefits for society and the climate overall. Emergency department visits and inpatient stays decreased over time and lung function declined during the study period as expected suggesting that remote care was not inferior to usual care during this period and within this population. Unfortunately, this “new normal” did not seem to stick as healthcare consumption patterns slowly returned to the “old normal”, indicating that remote healthcare was only a temporary replacement for “regular care”. Nevertheless, these findings provide prospects for the potential of remote monitoring for the future of CF care and for reducing healthcare related GHG emissions. This is especially relevant considering the Dutch health sector's contribution to the national climate change footprint is 7.3% of which private travel has a 5.3% share (16).

This study had several limitations. Firstly, selection bias could have occurred as mostly those pwCF with positive experiences might have participated in this study. However, we believe that our retrospective design will have reduced the impact of selection bias compared to prospective studies that might attract less pwCF from the onset of the study. This is strengthened by our earlier increased findings of negative psychosocial effects compared to other prospective studies (3). Moreover, our population consisted of a general CF population similar to the overall Dutch population spread across five of the seven Dutch CF centers which increases the generalizability of our findings. Nevertheless, factors that influence willingness and ability to participate in digital health interventions (e.g., digital health literacy) are not captured in standard demographics. It is therefore probable that for some pwCF outcomes could be less positive, which further emphasises the need for individualised approaches and innovative study approaches that address these differences (3). Secondly, this article did not study any associations between home monitoring frequency and GHG emissions or healthcare consumption but looked at overall differences between before and after introduction of the RMP. This is an important subject for future research to pinpoint what the basic requirements could be for remote care to replace physical care. Finally, we assumed that cars were used for private travel, but this might not always have been the case. Nevertheless, a reduction in any type of motorized travel and reductions in hospital resources would still be beneficial.

Safety for pwCF remains a concern when monitored at home instead of in clinic. Future research should include general CF cohorts assessed over a longer period of time and should also include more elaborate analyses of inpatient stays, emergency room visits, microbiological assessments and antibiotic therapy courses in order to better examine non-inferiority or superiority of remote care compared to physical care. These studies should pay particular attention to differences within subgroups based on broader determinants of digital health accessibility. Our study is in line with a recent systematic review that shows that private travel is the most important factor to reducing GHG emissions with remote care, especially in larger geographical areas where patients have to travel far to receive care (17). Although the Netherlands does not have a large geographical area, people with rare diseases, such as CF, usually need to travel further to specialised centres. However, this study also emphasises that there is a need for more comprehensive healthcare technology assessments on the co-benefits for climate change mitigation of remote care that include a broader range of indicators and parameters which will help to more accurately estimate actual GHG emissions (17).

In conclusion, remote monitoring of symptoms and lung function and teleconsultations in CF have the potential to reduce GHG emissions while maintaining the same quality of care. It is important to address these issues together to create political will for the implementation of digital health that empowers patients and HCP but also benefits society overall through aiding climate change mitigation.

## Data Availability

The raw data supporting the conclusions of this article will be made available by the authors, without undue reservation.
